# Establishing the Effect of Brushing and a Day’s Diet on Tooth Tissue Loss in Vitro

**DOI:** 10.3390/dj4030025

**Published:** 2016-08-09

**Authors:** Claire Forbes-Haley, Siân Bodfel Jones, Maria Davies, Nicola X. West

**Affiliations:** 1 UHBristol, Bristol Dental Hospital, Lower Maudlin Street, Bristol BS1 2LY, UK; 2 Clinical Trials Unit, School of Oral and Dental Sciences, University of Bristol, Lower Maudlin Street, Bristol BS1 2LY, UK; S.b.jones@bristol.ac.uk (S.B.J.); Maria.Davies@bristol.ac.uk (M.D.); n.x.west@bristol.ac.uk (N.X.W.)

**Keywords:** erosion, daily diet, tooth tissue loss

## Abstract

To develop an in vitro model to mimic the effects of meals equivalent to a day’s diet on tooth tissue loss (TTL). To identify how diet effects tooth wear and to test the efficacy of dental products designed to reduce tooth wear in a more realistic environment. A typical Friday diet was devised comprising: Breakfast then brushing, lunch, dinner then brushing. Groups of enamel samples were exposed to one meal, or all three in series, a control group was exposed to water and brushed. The daily cycle was repeated to represent two days’ consumption; TTL was quantified by non-contact profilometry. This pilot study highlighted adaptions that could be made to the model such as human enamel and saliva to further replicate natural eating habits. The sum of the TTL measured after Breakfast, lunch and dinner (bovine enamel specimens exposed to single meals) was less than that exhibited by the group of samples exposed to the series of meals but this difference was not significant (*p* = 0.09).In the absence and presence of brushing, TTL caused by breakfast and dinner was similar, but significantly greater than that caused by lunch (*p* < 0.05). While brushing increased TTL, this increase was not significant. It is possible to model a daily diet in vitro, and the data obtained confirms that the combination of food and drink affects the degree of TTL. This supports the further development of an in vitro model that includes alternative foodstuffs. This would aid understanding of the effects different diets have on TTL and could test new products designed to prevent TTL.

## 1. Introduction

Tooth tissue loss (TTL) is a common condition and is a pathological, non-carious irreversible loss of tooth tissue, often having a multifactorial aetiology [1]. Studies have shown that the early stage of tooth wear can be recognised by the loss of surface enamel and by the softening of the underlying enamel tissue leaving it susceptible to further tooth wear from abrasion and continued erosion [2]. Tooth brushing and coarse foods are abrasives that can contribute to further TTL, especially when in combination with an erosive diet [3]. Human enamel is thus subjected to a plethora of erosive and abrasive insults on a daily basis and dental erosion is a condition which is steadily growing [4].

Tooth wear is multifactorial with aspects of erosion, attrition and abrasion, but the contribution of erosion to tooth wear may be increasing [5]. Tooth surface loss can be complex and requires interdisciplinary long-term management, stressing the liaison between dental professionals to aid prevention and maintain oral hygiene. Identification of the etiology of tooth tissue loss helps with effective management but can be very difficult to accurately ascertain [6]. The Royal College of Surgeons’ guidelines, 2013 encourage questioning each patient about their medical history and medication to ascertain risk factors. The guideline also highlights that dietary intake should be assessed with careful questioning on the intake of specific items, and it is suggested that dentists ask patients to record a three-day diet history to include a weekend and the times of food/drink consumption. Dietary counseling that aims to reduce the intake of products that can exacerbate tooth wear needs to be tailored to the individual so can only be provided with any accuracy after diet assessment. The Royal College of Surgeons suggest future research may establish relationships and the influence of co-factors in the erosive process, this study’s model could be used to aid this research [7].

What is considered to be an acceptable degree of TTL is dependent upon the anticipated life span of the tooth, whether it is deciduous or permanent and the relative to the age of the patient. When TTL has been aggressive enough that a restoration is required this results in a lifetime of restorative interventions, with associated implications in terms of time and finance [8]. This is why further understanding into TTL, causative agents and prevention is important to the population as more people are keeping their teeth for longer [9]. Risk factors and food interactions need to be understood to allow successful prevention. Understanding of people’s diets and their brushing regimes are an important part of this prevention.

It is well known that dietary substances such as soft drinks and abrasive foods cause TTL when investigated independently [10] but limited research has investigated how a series of acidic and abrasive challenges that occur during a normal day of eating and drinking affect the amount of TTL. Experiments to test how effectively toothpastes prevent/reduce TTL also rarely incorporate the erosive and abrasive effects of a food and drink sequence. Toothpastes containing fluoride have been shown to help reduce and prevent TTL [11] and Ganss et al. [12] using a single erosive agent showed that treatments with fluoridated toothpaste (26 μmol/L·F^−^) could significantly reduce tooth erosion by 50% to 90% on enamel and 10% to 55% on dentine. However more realistic models representing a series of meals and twice daily brushing are limited. More realistic models could better identify aggressive foods effecting TTL and test the efficacies of toothpastes developed to prevent TTL.

In the present study diet diaries were used to determine a diet that could be tested in vitro. As the diet data collected on a Friday showed a combination of habits from weekday and weekend activities the average “Friday diet” was chosen and replicated for the study Common meals recorded from the Friday diets in a population of dental patients in the South West of England were used as the basis for the diet. Items such as snacks and alcohol were included in this diet, to reflect the normal habits of this population seen in their diet diaries.

The aim of this study was to develop an in vitro model containing a series of erosive and abrasive insults representing a daily diet, combined with a typical oral care routine (twice daily tooth brushing) that could be used to better test the efficacy of oral care products designed to reduce TTL. Difficulties in preparing foodstuffs, and the application of foods and drinks in a way that would mimic oral consumption were assessed. For comparison, the individual amount of TTL following exposure to single meals and that obtained following exposure to the meals in series for each day was also investigated.

## 2. Results

As part of this pilot we wanted to test whether a daily diet could/should be used instead of individual foodstuffs and for testing toothpastes. During testing it highlighted the difficulties with using dry food stuffs such as peanuts. It is hard to achieve continued contact onto bovine enamel specimens with dry food. The addition of human saliva would reduce some of the challenges of dry food.

**Figure 1:**
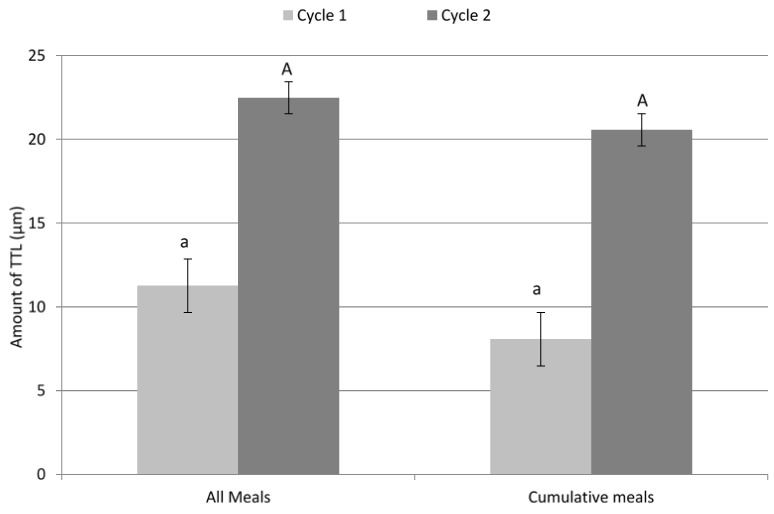
The total amount of tooth tissue loss (TTL) caused by a sequence of meals compared to the sum of individual meals following one and two cycles. Error bars show standard deviation and the use of different letters signifies that groups are significantly different (*p* < 0.05). Lower case letters are associated with cycle 1 and upper case letter are associated with cycle 2.

Bovine enamel specimens exposed to the series of meals in a ‘Friday’ diet exhibited greater TTL than the sum of TTL measured for samples exposed to the individual meals (22.47 µm and 20.61 µm respectively) this was also seen following two cycles (2nd day) but this result was not significantly different (*p* = 0.09; Figure 1).

**Figure 2:**
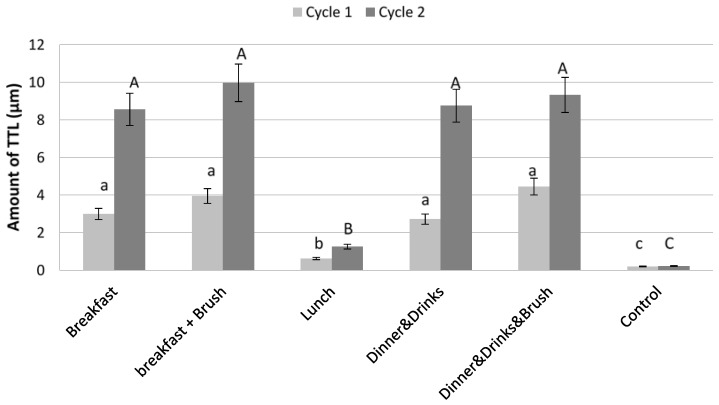
The amount of TTL caused by individual meals following one and two cycles. Error bars show standard deviation and the use of different letters signifies that groups are significantly different (*p* < 0.05). Lower case letters are associated with cycle 1 and upper case letter are associated with cycle 2.

Considering breakfast and dinner, the results from the single meals (Figure 2) showed that the amount of TTL measured following dinner and brushing was statistically similar to TTL following breakfast and brushing after both cycle 1 (*p* = 0.244) and 2 (*p* = 0.151). Following Day 1 and Day 2 cycles, the amount of TTL caused by lunch was significantly less than that caused by the other meals (*p* = 0.043) and this ‘lunchtime meal’ caused the least amount of TTL (0.63 µm following the first cycle and 1.26 µm following the second cycle) despite the Coca-Cola^®^ (Atlanta, GA, USA) drink having one of the lowest pH of all foodstuffs. The difference in TTL between Breakfast and Breakfast & Brush (*p* = 0.263) also Dinner & Drinks and Dinner & Drinks & Brush (*p* = 0.136) was not shown to be significant. Control samples exposed to water and brushing showed little TTL. The difference in TTL for control samples between different meals with the higher TTL levels, Breakfast and Breakfast & Brush and Dinner & Drinks and Dinner & Drinks & Brush was not significant (*p* = 0.217, *p* = 0.247). Control samples total TTL was significantly different from all meals on tested samples (Total: 0.012 µm cycle 1, 0.029 µm cycle 2, and *p* = 0.024).

## 3. Discussion

*Erosion and Abrasion*: To date, dental erosion literature focuses mainly on the erosive potential of beverages due to their frequent consumption, contact time with the hard tooth tissue and subsequent proven link to tooth wear [13]. However, there are studies that have looked at the erosive potential of foodstuffs, for example which have demonstrated the erosive nature of roasted vegetables as compared to vegetable prepared for consumption in other ways [14]. Distinctive patterns of tooth wear can also be caused by certain foodstuffs containing, abrasive dietary matter, most evidence from this comes from studies of teeth from our ancestors but similar patterns are still observed today and have been associated, for example with chapattis flour products [15].

The abrasive effects of tooth brushing [16] and oral soft tissues [2] can also result in tooth tissue loss, this can be further exacerbated after enamel or dentine surfaces have been softened by dietary acid intake. Exposure to acidic foodstuffs combined with brushing in a repeated cycle challenges the softened surface of enamel and can even remove this surface layer. Once this most protective outer layer of enamel has been damaged the sub-surface is more susceptible to acid attack [17]. A softened surface layer can be removed by very gentle actions and it has been suggested chewing alone can add enough abrasion to increase overall TTL [18].

The present study explored the erosive and abrasive effects of many dietary agents on enamel tooth tissue loss following simulated “meals” consumed in a day, with a twice-daily brushing regimen. This was with the aim of developing an in situ model that recreates normal TTL that could be used to test products designed to protect against TTL and to highlight any food combinations, which are more aggressive.

Average TTL in a year has a great range from 10 to 200 μm as patient habits and foods are highly variable. An estimate 60 μm can be used as an average as seen in control subjects [19]. The total erosion recorded in this study was between 20 and 25 μm. This implies that the insults in this in vitro model were more aggressive than average as this value would relate closer to 3 months of TTL. The high level of toothwear observed may reflect the fact that other factors such as buffering from saliva, natural cleansing, mastication and movement of food from the tongue in the mouth are present in vivo and could reduce the total TTL. These factors should be considered when using this model for other studies especially the inclusion of human saliva.

*Food exposures:* Methods used for food preparation and exposures were designed in consideration of normal mastication of different types of food stuffs. The length of time for solution immersion and food exposure was decided from assessment of three subjects eating the same meal plan as the “Friday” diet. Differences were seen in length of time spent chewing and the frequency of drinking among subjects but an average value was used in this study. The types of preparation attempted to replicate the normal environment; this is why different methods of food preparation were used for different foods. Difficulties were encountered during piloting of exposure to dryer food stuffs this is why bovine enamel specimens were secured using sticky wax. It was felt important to expose to dry food and not to add moisture making them smoother as that the attrition from the food would be better represented. In the mouth dry foods would tend to stimulate saliva and in further studies addition of saliva to the food at intervals could be considered. Alternatively bovine enamel specimens could have been present in the pestle and mortar while the food was being pulverized but this would have compromised the specimens. Food could have been placed in the CAT stirrer without being pulverized and then exposed to bovine enamel specimens. From piloting studies it was found that the action of the CAT stirrer on bovine enamel specimens pushed large pieces of food to the sides of the container or displaced specimens and then no contact was achieved. People eat and drink at the same time and at this stage of the model design it would have been difficult to replicate this as mixing of food/liquids would be required.

For some groups bovine enamel specimens were measured for TTL after each meal. For other groups bovine enamel specimens were measured for TTL once the specimens had been exposed to all meals. There was a possibility that repeated exposure of bovine enamel specimens may yield greater over all TTL than exposure to one meal alone. This would mean that all samples would have to run through each meal in turn to accurately calculate total TTL for a daily diet. Therefore, the total TTL for a daily diet would not be the same as the sum of TTL after each meal. The results showed there was a marginal difference in the total TTL for single meals, cycle 1:8.07 μm cycle 2:20.6 μm and the total for the cumulative meals cycle 1:11.26 μm cycle 2:22.47 μm. However, the difference between single or multiple exposures and the difference between cycles was not significant *p* = 0.09, *p* = 0.08 respectively.

The lack of significant difference between single or multiple exposures would allude to the idea that the total TTL could be found by; either exposing bovine enamel specimens to individual meals (combinations of food and drink) or by the sum of individual food/drink exposures in combinations that could make a meal, giving the same total TTL. Therefore, the total TTL from exposure to food and drinks mimicking meals could give an impression of the potential total TTL from a normal diet (multiple meals). Bovine enamel specimens could be run through common food and drink combinations (meals), these different meals could then be used in different combinations. This in turn highlighting which combinations are more or less aggressive, without having to run all bovine enamel specimens though a whole day’s diet, thus reducing the testing process. These meal combinations could then be manipulated to more appropriately highlight aggressive food/drinks/agents and test potential preventative mechanisms for TTL.

Work carried out by Bartlett et al. [20] recognised that in some parts of the UK it is quite common to consume spicy food followed by alcohol. Most western diets commonly contain alcohol and very few diets are devoid of processed foods [21,22].

*The diet and food combinations:* The diet chosen represented a relatively acidic, western diet, as determined from patient dietary records. This diet would vary in different populations and more diet diaries from a larger region would help to gain a more realistic picture of the average diet. Stratifying for age group and gender could enable a better sample of the population as a whole. Results considered the total TTL from single and a series of meals but it was shown that there was no statistical difference between these two methods of exposure. The results demonstrated tooth wear following each meal. Due to combinations of intake and exposure times chosen, results varied with TTL from “dinner” samples being similar to “breakfast” samples, and “lunch” samples showing far less wear despite the Coca-Cola^®^ drink having one of the lowest pH of all foodstuffs. Various reasons can be postulated to explain these results. Breakfast and dinner could potentially have been more erosive and abrasive than lunch due to the foodstuffs used. For breakfast, the erosive grapefruit juice with the abrasive and erosive toast and marmalade combine to act as an aggressive insult. Dinner consisted of curry with lager followed by cider and peanuts. The curried food and alcoholic beverages were all acidic and in combination with the abrasive effects of the peanuts provide another potentially aggressive insult. Lunch however consisted of pasta with tomato, basil and cheese with Coca-Cola Zero^®^. Even though cola can be very erosive, the pasta due to its consistency could have stayed on the surface of the bovine enamel specimens and with the cheese acted as protection against erosive insults. It could be postulated that a combination of some of the foodstuffs, such as casein found in cheese/dairy verses acidic drinks have conflicting effects, and could result in reduction or even inhibition of TTL. Grapefruit is known to be an erosive citrus fruit [23] and in comparison the addition of casein to products has shown to reduce erosive tissue loss in situ [24]. The abrasive foods in combination with acidic food however could work synergistically and increase the effects of TTL by removing a greater amount of tooth tissue. Brushing the bovine enamel specimens following breakfast and dinner meals also added to TTL. The amount of TTL evident after brushing the control samples was no greater than 0.035 μm. However, following softening of the surface caused by the erosive insults during both breakfast and dinner regimes, the amount of TTL induced by brushing was up to 1.846 μm highlighting how brushing immediately after a meal can add to an erosive challenge.

*Saliva:* Human saliva was not included in this treatment regimen, as this study hoped to review the use of the meal regimes and at this time did not fully attempt to replicate the oral environment. It is known that 30 min exposure to saliva can create a substantive pellicle that can reduce acid erosion [25]. A substantive pellicle and continual soaking in saliva would represent a more natural model. However, whether saliva would remineralise or harden the softened surface of enamel after acid challenge as delivered in the present study as part of a meal is unclear as no benefit to waiting for 2 or 4 h after erosive challenge before toothbrushing has been reported [26,27]. The saliva would also create an instant buffering effect against the acidic food challenges and could therefore have reduced the foodstuffs erosive potential [28]. If saliva had been used in this study it is likely that there would have been less TTL from brushing after meals and the total TTL would have been lower. The use of human saliva could be an addition in future studies.

*Brushing:* In the present study, a brushing regimen mimicked a twice-daily oral hygiene pattern, 2 s allocated to each sample per brushing sample. This was calculated on the basis of an individual brushing for a one minute brushing cycle [29], three surfaces of 28 teeth; buccal, lingual and occlusal. The force applied was standardised as was toothpaste concentration, brushing technique and model of brush. The results from this study showed no significant difference in TTL with brushing twice a day and a minimum of 1 h was left before/after food exposure, allowing an element of remineralisation [30]. However it has been suggested that the brushing frequency, brushing less than 1 h after an erosive insult, previous TTL and toothpaste are the culprits for additional TTL on teeth which already suffer TTL.

*Pilot design:* Due to the design of the model further changes could be made if required to take use of this model further and to better represent the oral environment. The use human enamel and saliva could be incorporated. Saliva could be used before and between meal exposures as well as addition to food stuff during exposure. Exposing bovine enamel specimens to mixtures of food and drink would further replicate people’s natural eating habits.

*Clinical Rationale for study:* In reality wear occurs from a number of sources, food, drink, oral tissues and brushing. This pilot model planned to better represent a natural diet sequence and exposure to foodstuffs and tooth brushing. This study shows how different foods and their combinations can affect TTL.

*Clinical applications:* This study highlights how important diet diaries and their assessment is when considering prevention and gaining understanding of individual patients potential aggravating factors for their TTL. With further development we hope this study model can be used to help identify any products, which may reduce TTL.

## 4. Materials and Methods

*Study design:* Diet diaries were collected from 30 randomly selected patients attending for treatment with undergraduates at Bristol Dental Hospital. As Friday diet data showed a combination of habits from weekday and weekend activities this was chosen as the day to replicate for the study and common meals recorded from Fridays were used as the basis for the diet. Items such as snacks and alcohol were included in this diet, to reflect the collected diet diaries.

Bovine enamel specimens, representing the tooth surface, were exposed to food intake and brushing as the teeth would come into contact with over a day. This study piloted the diet model and assessed the effect of fluoride toothpaste on the amount of TTL following a series of natural exposures.

*Sample preparation:* 25 flat bovine enamel specimens were prepared from permanent bovine teeth. Bovine enamel was sectioned using a water-cooled high-speed diamond saw (MicroSlice; Metal Research, Cambridge, UK) in 6mm square. Each section was mounted in epoxy resin (Stycast; Hitek Electronic Materials, Scunthorpe, UK) and polished using SiC discs (p1200) followed by SiC powder (p1200) and finished by polishing with Al_2_O_3_ powder (0.3 μm) as a suspension in deionised water to achieve a smooth, flat surface. Bovine enamel specimens were ultrasonicated in deionised water between each polishing stage to remove any debris. Each treatment group (control or test) was assigned 5 samples.

*Measurement techniques:* Non-contact white light profilometry (Proscan 2000, Scantron, Taunton, UK) was used to measure the amount of tissue loss. Initial scans were performed to record initial surface variations on the sample. Bovine enamel specimens with surface variations within ±1 μm over a 2 × 1 mm^2^ area were accepted for inclusion in the study. Following initial scanning, the ends of each bovine enamel specimen were protected with tape providing two control areas and ~1.5 mm wide enamel window. Following treatments, the tape was removed and the amount of surface loss measured using a three-point height measurement where the difference in height between the two control areas and the central treated area was measured [31].

*Sample brushing*: For brushing of the bovine enamel specimens each specimen was mounted onto a strip of perspex using sticky wax, 1 treatment group of 5 specimens were placed in a row spaced to avoid accidental brushing of two adjacent specimens. Brushing was performed using an electric toothbrush (Oral B Vitality Precision Clean Power toothbrush fitted with EB17 Oral B flex iSOFT toothbrush head, Procter and Gamble, Surrey, UK). Each test group and the control group were allocated different toothbrush heads to avoid contamination between groups, the heads were not renewed between brushes. A pea sized amount of toothpaste weighing between 1.1 g ± 0.1 g (Colgate Total, Colgate-Palmolive (UK), Surrey, UK) containing 1450 ppm·F^−^ (27.6 μmol/L·F^−^) was dispensed and applied by brushing in a back and forth motion across mounted bovine enamel specimens. Colgate^®^ Total was chosen for this study as it is an industry lead as a standard toothpaste in the UK and has been used in many other similar studies [32]. The bovine enamel specimens were brushed on a balance so that a constant force of ~200 g was applied for 2 s per specimen [33,34]. Control bovine enamel specimens were brushed under the same conditions. After each brushing any dentifrice slurry was removed from the bovine enamel specimens using deionised water then stored in deionised water. A minimum of 1 h elapsed after food exposure, before brushing and before further meal exposure.

*Dietary challenges*: Bovine enamel specimens were exposed to beverages, moist pulverised foodstuffs or dry foodstuffs. The length of exposure to beverages (equivalent to drinking time), and foods (equivalent to chewing time) was calculated from the average times recorded for three people who piloted the set ‘Friday’ diet plan. The ‘Friday’ diet was split into three stages representing breakfast (Pure Pink Grapefruit Juice with Bits (Sainsbury’s Supermarkets Ltd., London, UK) followed by toast (Allison Batch Wholemeal loaf, Allied Bakeries, ABF Grain Products Limited, London, UK) and Orange marmalade (Tiptree Wilkin and Sons Limited, Essex, England, UK)), lunch (Sainsbury’s Tomato and Basil Pasta (Sainsbury’s Supermarkets Ltd., London, UK) and Coca-Cola Zero^®^ (The Coca-Cola Company, Atlanta, GA, USA)) and dinner (Chicken Balti and Pilau Rice (Sainsbury’s Supermarkets Ltd, London, UK) with Stella Artois lager (Anheuser-Busch InBev, Leuven, Belgium) followed by mulled Bulmers original Irish cider (Bulmers Ltd. of Clonmel, Edinburgh, UK) and KP original salted peanuts (United Biscuits, Middlesex, UK). One group (*n* = 5) was exposed to all 3 stages whilst 3 groups were assigned separately to breakfast, lunch or dinner. This sequence of meals was repeated twice to represent 2 days’ worth of exposure. Bovine enamel specimens were brushed in toothpaste slurry following the breakfast and dinner regimes.

Some adaptation of the model design had to be made to allow sufficient simplicity for accurate repetition of the sequences of eating and drinking. For the drinks exposure, bovine enamel specimens were immersed in 200 mL of beverage and the specimens were stirred at an equivalent linear velocity of 0.25 m·s^−1^ using a CAT stirrer (CAT 100SD, Ingenieurbüro, M.Zipperer GmbH, Staufen, Germany) and the length of exposure is shown in Table 1. For the fresh foodstuffs 15 g were pulverised using a pestle and mortar for 5 s then exposed using the CAT stirrer as above. Exposure to toast was carried out by mounting the bovine enamel specimens in a row on a strip of perspex using sticky wax and rubbing the specimens against the food under a constant force of ~300 g, measured using a balance for 15 repeated strokes over 10 s. A minimum of 5 h was left between each meal exposure and bovine enamel specimens were brushed 1 h after meal exposure where indicated. At least 12 h was left between cycles of exposure whilst bovine enamel specimens were stored in deionised water. Bovine enamel specimens were exposed at the temperatures that the food and beverages would normally be consumed and the pH was measured at the temperature of exposure. Between each meal exposure bovine enamel specimens were rinsed for 30 s with deionised water.

*Measurement of TTL:* Non-contact profilometry was used to record TTL following breakfast, brushing after breakfast, lunch, dinner and brushing after dinner. Table 1 shows the food regimens used and their pH. Double lines represent where non-contact profilometry measurements were taken.

*Statistical analysis:* SPSS 19 software (IBM Corporation, New York, NY, USA) was used to analyse the data. The values following single meals were compared using one-way ANOVA followed by Tukey HSD post hoc test. Endpoint TTL was compared following the series of meals and the cumulative total of the individual meals using unpaired *t*-test. Statistically significant differences were quoted where *p* < 0.05.

## 5. Conclusions

This study shows that it is possible to recreate a model using a diet of food and drinks combined in a sequence that can elicit TTL. A series of dietary compounds for single meals has the same potential for TTL as a series of dietary compounds for multiple meals (cumulative), therefore either of these methods to measure TTL could be used without effecting the final results. Exposure to acidic and abrasive substances associated with a “Friday” diet has the potential to cause TTL. The extent of TTL cannot be predicted solely by the pH of the substance. This pilot model using a realistic meal sequence can be developed further to accurately highlight areas where prevention could be implemented to aid reduction of TTL.

## Figures and Tables

**Table 1:** The ‘Friday’ diet meal sequence showing pH at temperature and time of exposure. Double lines represent where non-contact profilometry measurements were taken.

Drink/Food	Temperature (°C)	pH	Time (min)
Breakfast			
Grapefruit juice	4	3.32	5
Marmalade and Toast	22	3.16	2
Grapefruit Juice	4	3.32	5
Brushing for 2 s/sample			
Lunch			
Roasted Vegetable Pasta	22	5.96	10
Cola	4	2.81	10
Dinner			
Lager	4	4.24	10
Chicken Balti curry	65	5.17	10
Lager	4	4.24	10
Mulled Cider	65	3.41	10
Dry Roasted peanuts	22	-	10
Mulled Cider	65	3.41	10
Brushing for 2 s/sample			
